# Local Concentrations of TGF-β1 and IGF-1 Appear Determinant in Regulating Bone Regeneration in Human Postextraction Tooth Sockets

**DOI:** 10.3390/ijms24098239

**Published:** 2023-05-04

**Authors:** Maria B. Asparuhova, Dominic Riedwyl, Ryo Aizawa, Clemens Raabe, Emilio Couso-Queiruga, Vivianne Chappuis

**Affiliations:** 1 Laboratory of Oral Cell Biology, Dental Research Center, School of Dental Medicine, University of Bern, Freiburgstrasse 3, 3010 Bern, Switzerland; dominic.riedwyl@unibe.ch (D.R.); r-aizawa@dent.showa-u.ac.jp (R.A.); 2 Department of Oral Surgery and Stomatology, School of Dental Medicine, University of Bern, Freiburgstrasse 7, 3010 Bern, Switzerland; clemens.raabe@unibe.ch (C.R.); emilio.couso@unibe.ch (E.C.-Q.); vivianne.chappuis@unibe.ch (V.C.); 3 Department of Periodontology, School of Dentistry, Showa University, 2-1-1 Kitasenzoku, Ohta-ku, Tokyo 145-8515, Japan

**Keywords:** osteogenesis, wound healing, growth factors, extracellular matrix proteins, cyclic strain, matrix stiffness, connective tissue grafts, transcription, implant placement, bone and soft tissue augmentation

## Abstract

Healing after tooth extraction involves a series of reparative processes affecting both alveolar bone and soft tissues. The aim of the present study was to investigate whether activation of molecular signals during the healing process confers a regenerative advantage to the extraction socket soft tissue (ESsT) at 8 weeks of healing. Compared to subepithelial connective tissue graft (CTG), qRT-PCR analyses revealed a dramatic enrichment of the ESsT in osteogenic differentiation markers. However, ESsT and CTG shared characteristics of nonspecialized soft connective tissue by expressing comparable levels of genes encoding abundant extracellular matrix (ECM) proteins. Genes encoding the transforming growth factor-β1 (TGF-β1) and its receptors were strongly enriched in the CTG, whereas the transcript for the insulin-like growth factor-1 (IGF-1) showed significantly high and comparable expression in both tissues. Mechanical stimulation, by the means of cyclic strain or matrix stiffness applied to primary ESsT cells (ESsT-C) and CTG fibroblasts (CTG-F) extracted from the tissue samples, revealed that stress-induced TGF-β1 not exceeding 2.3 ng/mL, as measured by ELISA, in combination with IGF-1 up to 2.5 ng/mL was able to induce the osteogenic potential of ESsT-Cs. However, stiff matrices (50 kPa), upregulating the TGF-β1 expression up to 6.6 ng/mL, caused downregulation of osteogenic gene expression in the ESsT-Cs. In CTG-Fs, endogenous or stress-induced TGF-β1 ≥ 4.6 ng/mL was likely responsible for the complete lack of osteogenesis. Treatment of ESsT-Cs with TGF-β1 and IGF-1 proved that, at specific concentrations, the two growth factors exhibited either an inductive-synergistic or a suppressive activity, thus determining the osteogenic and mineralization potential of ESsT-Cs. Taken together, our data strongly warrant the clinical exploration of ESsT as a graft in augmentative procedures during dental implant placement surgeries.

## 1. Introduction

Following tooth extraction, socket healing involves dimensional ridge alterations of the local alveolar bone and soft tissues [1,2] as well as internal alterations resulting in bone formation within the socket [3,4]. The chronological sequence of events characterizing the extraction socket healing process has been described in a number of preclinical [5,6,7] and clinical studies [8,9,10,11]. In brief, due to hemorrhage during the tooth extraction, a fibrin clot is formed within 3 to 6 h, followed by infiltration of inflammatory cells within 2–3 days. The combination of inflammatory cells, vascular sprouts, and immature fibroblasts forms granulation tissue, which fully replaces the fibrin clot within 7 days [8]. After 20 days, most of the granulation tissue is replaced by a provisional connective tissue, the so-called provisional matrix, and bone formation starts at the apical region and the wall of the extraction socket. In human specimens obtained between 6 and 8 weeks of healing, the provisional matrix and immature woven bone are shown to occupy 60 and 35% of the tissue sample, respectively [3]. The two tissues are dominant in the extraction socket even at 12–24 weeks of healing [11]. Via a process of corticalization [12], the initially formed woven bone that lacks load-bearing capacity is gradually replaced by bone marrow and lamellar bone that forms a crestal hard tissue bridge. The complete remodeling of the woven bone into lamellar bone may take several months or years [3,11]. This long remodeling phase is significantly influenced by mechanical forms of stress [13]. The tissues in the oral cavity are subjected to a wide variety of mechanical forces, including compression, elongation, friction, shear, and hydrodynamic forces generated during mastication, speech, tooth brushing, and saliva flow [14,15].

The concept of immediate implant placement, when implants are placed in the fresh extraction socket right after tooth extraction, has become popular among patients and clinicians because it reduces the number of surgical interventions and treatment time. For many years, it was assumed that the immediate implant placement would prevent the three-dimensional ridge alterations of the extraction socket. Preclinical [16] and clinical [17,18] studies failed to confirm this assumption. Moreover, a fivefold higher risk of wound healing complications and an inadequate esthetic result with insufficient hard and soft tissue regeneration in 42% of the cases was evident [18]. Recent data have shown that implants placed in fresh extraction socket sites present statistically significant higher risk of failure than implants placed in healed extraction sockets [19,20], suggesting a positive impact of the healed microenvironment on the osseointegration of the implant. Indeed, the extraction socket healing process is locally orchestrated by a number of signaling molecules, such as the platelet-derived growth factors (PDGFs), transforming growth factor-β (TGF-β), fibroblast growth factors (FGFs), insulin-like growth factors (IGFs), bone morphogenetic proteins (BMPs), and others [3,21,22,23,24]. In a time- and space-dependent manner, these factors influence the recruitment of cells, their growth, and differentiation in the healing microenvironment. The initiation of hemostasis ultimately results in the release of PDGFs, TGF-β, FGFs, and IGFs by activated platelets [22]. Early activation of TGF-β and FGF-2 modulates the activation of resting fibroblasts and their proliferation, which subsequently contributes to the synthesis and maturation of the extracellular matrix (ECM) and organization of the granulation tissue [25]. In a rabbit model, it has been observed that, after initial high expression at early time points, FGF-2 assumed a relatively constant and low expression level between 2 and 8 weeks post extraction [26]. TGF-β showed a mild trend of increased expression at early time points whereas BMP-2 rose steadily throughout the investigated 8 weeks of healing, thus paralleling the observed increase in bone formation. Histological analyses of human extraction socket biopsies performed by Trombelli et al. have demonstrated increased density of BMP-7-positive cells in the early healing phases, with a clear tendency of decrease at 12–24 weeks post extraction [3]. Immunohistochemical investigation in a mouse model has reported the secretion of TGF-β by M2-like macrophages in the early inflammatory stage of socket healing, which stimulated osteoprogenitor cell proliferation and early differentiation [27].

For achieving successful, aesthetically pleasant and long-lasting implant-supported oral rehabilitation, both bone and soft tissue architecture of natural dentitions need to be adequately regenerated. To restore horizontal bone dimensions, simultaneous or staged guided bone regeneration (GBR) might be used alone or in combination with palatal connective tissue grafts (CTG), which are utilized for increasing the volume and improving the texture of the peri-implant soft tissues [28]. However, invasive tissue augmentation procedures utilizing CTGs increase the patient morbidity, the total surgical time, and, most importantly, the risks of peri- (e.g., damaging the palatal artery) and postoperative complications [29]. Presumably, the highly underinvestigated extraction socket soft tissue (ESsT) might share characteristics of both soft and hard tissue. Therefore, the aim of the present study was set as twofold: (1) to investigate how the ESsT, following 8 weeks of healing, differs from a palatal CTG and (2) to suggest, from a biological point of view, whether and how the ESsT, which is normally discarded at the time of implant placement, can be potentially utilized in an augmentation procedure. An interesting interplay between the TGF-β1 and IGF-1 growth factors, due to their specific local concentrations in the postextraction sockets at 8 weeks of healing, was evident. Greater knowledge about the factors in the ESsT that might influence the healing process can serve as a foundation for therapeutic alternatives, addressing clinically challenging situations that compromise the soft tissue healing as well as the osseointegration of dental implants.

## 2. Results

### 2.1. Strongly Increased Expression of Osteogenic Differentiation Markers in ESsT Compared to Subepithelial Palatal CTG

**Figure 1:**
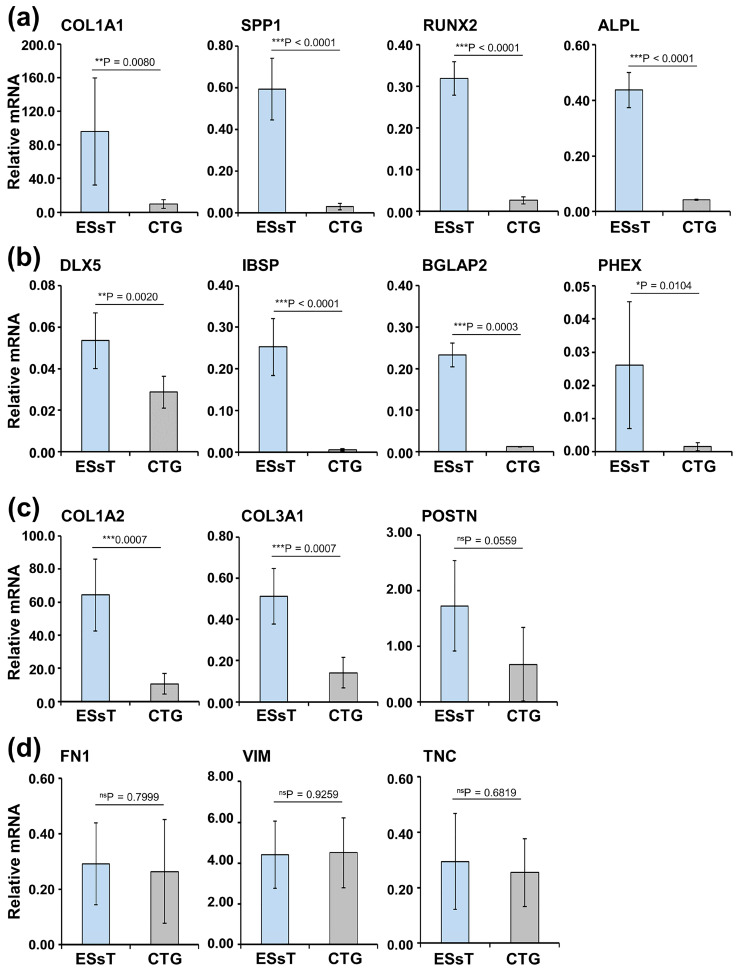
Expression of osteogenesis-related marker genes (**a**,**b**) and genes encoding ECM proteins (**c**,**d**) in ESsT and CTG samples. An ESsT and a CTG sample were harvested from the same patient and stored in RNAlater for 18–20 h before tissue homogenization, Proteinase K-treatment, and RNA extraction. Total RNA was subsequently used in qRT-PCR analyses of (**a**) COL1A1, SPP1, RUNX2, ALPL, (**b**) DLX5, IBSP, BGLAP2, PHEX, (**c**) COL1A2, COL3A1, POSTN, (**d**) FN1, VIM, and TNC transcripts normalized to GAPDH in the ESsT and CTG samples. Data represent means ± SD for 6 patients and significant differences between the two groups, *** *p* < 0.001, ** *p* < 0.01, * *p* < 0.05, ns = not significant are shown.

ESsT samples at 8 weeks of healing were characterized, in comparison with CTGs, for the expression of (a) osteoprogenitor and pre-osteoblast markers, such as COL1A1 (encoding the ECM protein collagen 1 type 1), SPP1 (encoding the ECM protein osteopontin), RUNX2 (encoding the Runt-related transcription factor 2), and ALPL (encoding alkaline phosphatase) (Figure 1a), and (b) osteoblast markers, such as DLX5 (encoding the distal-less homeobox 5), IBSP (encoding the integrin-binding sialoprotein), BGLAP2 (encoding osteocalcin), and PHEX (encoding the phosphate regulating endopeptidase homolog X-linked) (Figure 1b).

The ESsT samples exhibited an extremely strong and significant (*p* < 0.001) enrichment in the range of 10.4–45.0-fold of all osteogenic factors tested, except for COL1A1, DLX5, and PHEX (Figure 1a,b). However, the expression of the latter osteogenic markers was also increased either very significantly (*p* = 0.008 and *p* = 0.002 for COL1A1 and DLX5, respectively) or significantly (*p* = 0.01 for PHEX) by 1.9–16.7-fold compared to their expression in the CTGs (Figure 1a,b). The clearly upregulated expression of genes characterizing both pre-osteoblasts and mature osteoblasts in the ESsT compared to the CTG samples suggests that, at 8 weeks of healing, the bone formation process in the healing extraction socket has already been initiated.

Unlike the properties of epithelial, muscle, or nerve tissues, which are dependent on their cellular composition, the properties of the connective tissues are primarily determined by the type, quantity, and arrangement of their ECM. Therefore, we have further characterized the expression of several genes encoding ECM proteins abundant in connective tissue in general. Among them are COL1A2 (encoding the alpha-2 chain of collagen type I), COL3A1 (encoding the alpha-1 chain of collagen type III), POSTN (encoding periostin), FN1 (encoding fibronectin), VIM (encoding vimentin), and TNC (encoding tenascin-C) (Figure 1c,d). Similar to the osteogenic differentiation markers, the two newly tested collagen transcripts appeared extremely significantly enriched by 3.6–6.0-fold in the ESsT compared to the CTG (Figure 1c), whereas the remaining ECM protein-encoding genes were either slightly but not significantly enriched in the ESsT (see POSTN in Figure 1c) or equally abundant in the two tissue types (Figure 1d).

Thus, the restricted molecular characterization of the ESsT and CTG tissues performed in our study suggests that, in spite of the fact that the two tissue types share characteristics of nonspecialized soft connective tissue, the ESsT appears very distinct by exhibiting some features of specialized hard connective tissue.

### 2.2. Significantly Increased Expression of Genes Encoding TGF-β1 and Its Receptors in Subepithelial Palatal CTG Compared to ESsT Coincides with High Expression of IGF1 Transcript in Both Tissues

**Figure 2:**
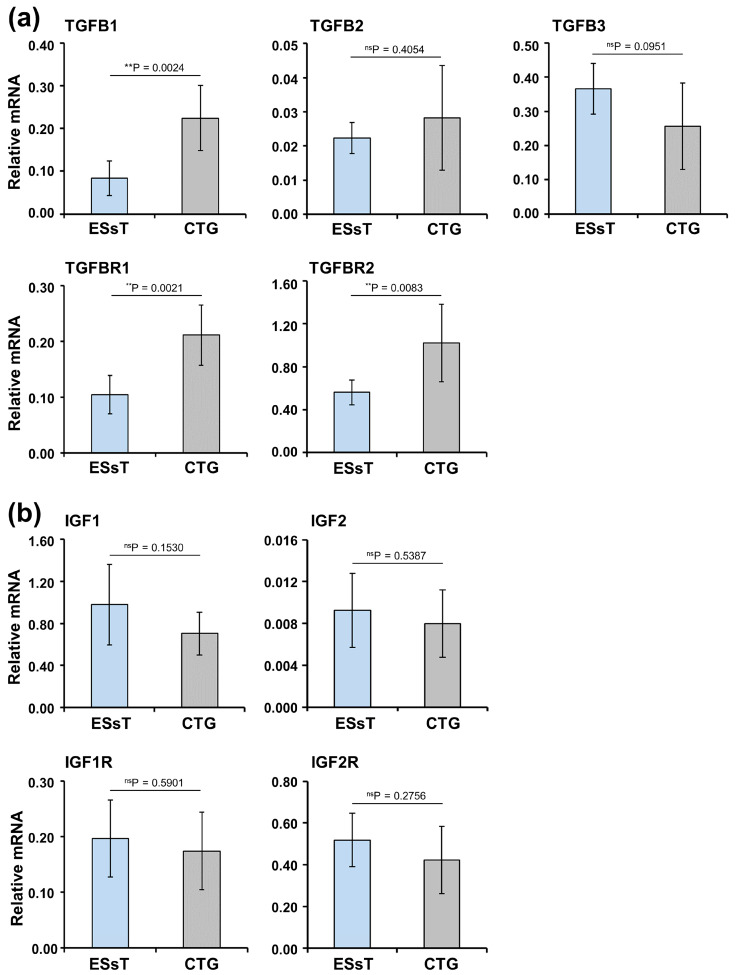
Significantly increased expression of genes encoding TGF-β1 and its receptors in subepithelial palatal CTG compared to ESsT coincides with high expression of IGF1 transcript in both tissues. qRT-PCR analyses of (**a**) TGFB1, TGFB2, TGFB3, TGFBR1, and TGFBR2 and (**b**) IGF1, IGF2, IGFR1, and IGFR2 transcripts normalized to GAPDH in the ESsT and CTG samples. Means ± SD for 6 patients and significant differences between the two groups, ** *p* < 0.01, ns = not significant are shown.

To investigate further the similarities and, respectively, the differences between ESsT and subepithelial palatal CTG, we analyzed the expression of genes encoding different isoforms of TGF-β, IGF, FGF, and BMP and their respective receptors in the two tissue types. The chosen growth factors play an important role in regulating both osteogenesis and oral soft tissue healing [30,31]. Compared to the ESsT group, the CTG group showed strong enrichment in TGFB1 (encoding the TGF-β1 isoform), TGFBR1 (encoding the TGF-β1 receptor I, also known as activin A receptor type II-like kinase), and TGFBR2 (encoding the TGF-β1 receptor II) transcripts by 2.7-, 2.0-, and 1.8-fold, respectively (Figure 2a). The expression levels of the TGFB2 and TGFB3 transcripts, encoding the TGF-β2 and -β3 isoforms, did not differ significantly between the two tissues.

Compared to the low expression (Ct > 28) of genes encoding isoforms of FGF and BMP (Supplementary Material, Figure S1) and their respective receptors, IGF1 (encoding the IGF-1 isoform) was the only transcript expressed at considerably high levels (Ct = 20–21) in both ESsT and CTG, despite the not significant difference in its expression between the two tissue types (Figure 2b). The IGF2, encoding the IGF-2 isoform, as well as the IGF1R and IGF2R, encoding two receptors for the IGF ligands, showed lower and not significantly different expression in the two tissue types (Figure 2b).

Thus, our data pointed to TGF-β1 as the only growth factor, among the four families of growth factors tested, exhibiting differential expression in the ESsT and CTG tissues and IGF-1 as the only growth factor with a significantly high expression in both tissues.

### 2.3. Cyclic Strain Applied to Primary Mesenchymal Cells Originating from ESsT and CTG Tissues Induces TGF-β1 and IGF-1 Expression Reflecting the Differences in the Growth Factor Gene Expression Observed at a Tissue Level

**Figure 3:**
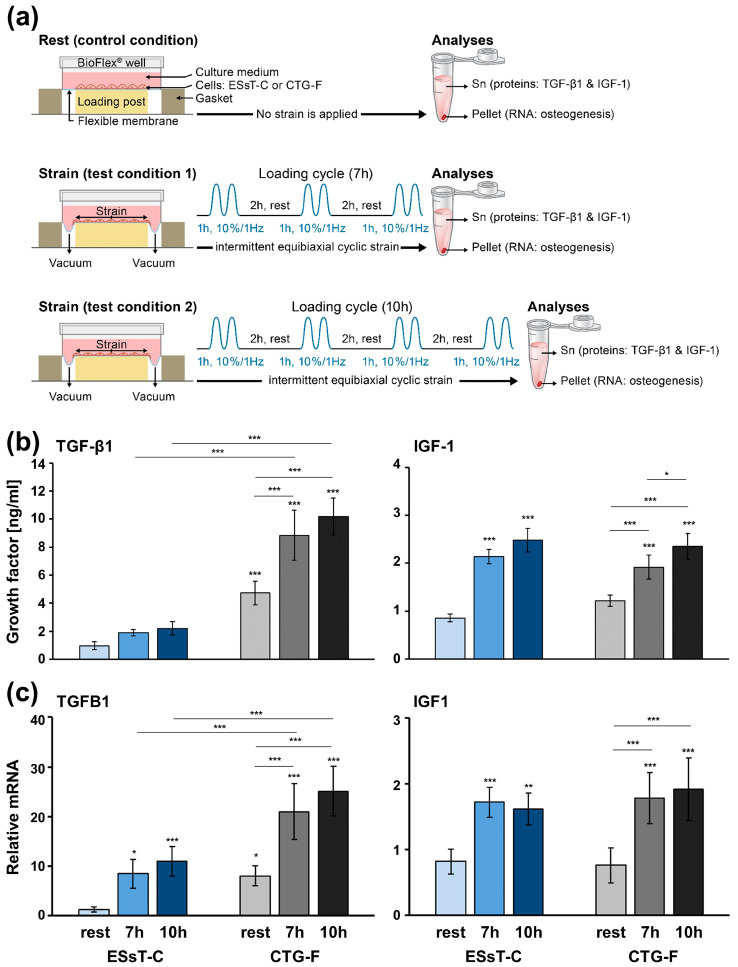
Cyclic strain applied to primary mesenchymal cells originating from ESsT and CTG tissues induces TGF-β1 and IGF-1 expression reflecting the differences in the growth factor gene expression observed at a tissue level. (**a**) Schematic representation of culture conditions. ESsT-Cs and CTG-Fs were cultured on fibronectin-coated silicone membranes in BioFlex^®^ culture plates in 0.3% serum-containing medium for 24 h before applying intermittent equibiaxial cyclic strain according to the loading cycles depicted under test conditions 1 and 2. In brief, the cells of the two cell types were either left at rest (control condition) or subjected to a 7- or 10-h loading cycle consisting of 1-h cyclic strain with 10% amplitude at a frequency of 1 Hz, alternating with 2-h rest intervals for a total of 7 or 10 h, respectively. Cell culture supernatant and pelleted cells were used in subsequent analyses at a protein and mRNA level, respectively. (**b**) Analyses of TGF-β1 and IGF-1 protein content in culture supernatants of ESsT-Cs and CTG-Fs at rest or after cyclic strain application, as indicated in (**a**). Values normalized to DNA content, to compensate for potential differences in the cell proliferation rate, are expressed relative to the values of control ESsT-Cs at rest. Data represent means ± SD from six independent experiments performed with primary ESsT-C and CTG-F cells from six different donors. Significant differences to the ESsT-Cs at rest unless otherwise indicated, *** *p* < 0.001, ** *p* < 0.01, * *p* < 0.05 are shown. (**c**) qRT-PCR analyses of TGFB1 and IGF1 mRNA levels. Values normalized to GAPDH are expressed relative to the values of resting ESsT-Cs. Data and statistical significance are presented as in (**b**).

To reproduce a physiological environment, in which oral tissues are constantly subjected to mechanical stimulation during chewing, speaking, tooth brushing, saliva flow, or surgical interventions [14,15], we subjected primary mesenchymal cells originating either from the ESsT, hereafter called ESsT-cells (ESsT-C), or from the CTG, called CTG-fibroblasts (CTG-F), to intermittent equibiaxial cyclic strain as schematically illustrated in Figure 3a. Two strain conditions, depicted as 7- and 10-h loading cycles, respectively, were applied. The 7-h loading cycle consisted of three 1-h cycles of 10% strain at 1 Hz, alternating with 2-h rest (no strain) intervals. Similarly, the 10-h loading cycle included four 1-h strain cycles alternating with 2-h rest intervals after the first, second, and third strain cycles, respectively. Immediately after the last strain cycle, TGF-β1 and IGF-1 proteins were quantified in the cell culture supernatant whereas cells were pelleted for subsequent gene expression analyses.

ESsT-Cs and CTG-Fs subjected to the two strain conditions showed no significant changes in viability and proliferation compared to control cells not subjected to cyclic strain (Supplementary Material, Figure S2). However, the mechanically stimulated cells exhibited slightly increased stress fiber formation compared to cells at rest, as seen by F-actin stain shown in the Supplementary Material, Figure S3. The TGF-β1 protein secreted by resting ESsT-Cs measured 0.97 ± 0.28 ng/mL versus 4.74 ± 0.84 ng/mL in resting CTG-F cells (Figure 3b). Cyclic strain applied with the 7- and 10-h loading cycles caused a gradual 1.9–2.3-fold increase in the TGF-β1 protein released by each of the two cell types. However, in the supernatant of strained ESsT-Cs, the TGF-β1 concentration did not exceed 2.21 ± 0.47 ng/mL (at loading cycle 10 h) and, thus, appeared not significantly different from the ESsT resting control. In contrast, the approximately fivefold higher concentration of TGF-β1 released by CTG-Fs compared to ESsT-Cs remained after the cyclic strain application, increasing it to 10.18 ± 1.30 ng/mL (at loading cycle 10 h), and, thus, appeared significantly different from the resting controls as well as from the strained ESsT-Cs.

In contrast, the quantity of IGF-1 protein secreted by resting ESsT-Cs and CTG-Fs did not differ and amounted to 0.86 ± 0.08 and 1.22 ± 0.12 ng/mL, respectively (Figure 3b). However, the application of cyclic strain gradually and extremely significantly increased the IGF-1 concentration up to 2.9-fold for strained ESsT-Cs and up to 1.9-fold for strained CTG-Fs compared to the respective controls at rest.

In contrast to TGF-β1, whose release from CTG-Fs remained significantly higher than the release from ESsT-Cs under all three conditions, the IGF-1 protein release, despite being induced by cyclic strain, remained unchanged between the two cell types under all tested conditions. Furthermore, the analyses of TGFB1 and IGF1 mRNA levels in resting and cyclically strained ESsT-C and CTG-F cells fully reflected the results obtained at a protein level (Figure 3c).

In conclusion, the intermittent equibiaxial cyclic strain application, which was designed to mimic the mechanical irritation of oral tissues during chewing, speaking, tooth brushing, or surgical interventions, appears to significantly stimulate the expression of TGF-β1 and IGF-1 above their expression levels in cells at rest. That way, each of the three conditions tested (rest and 7- and 10-h intermittent cyclic strain) reflects the expression of the two growth factors observed at a tissue level, namely higher TGF-β1 expression in CTG compared to ESsT and high but not significantly different expression of IGF-1 in both tissues. Therefore, the cyclic strain application to cultured cells originating from each of the two tissue types appears a suitable approach to further investigate the potential regulation of osteogenesis by TGF-β1 and IGF-1 in the ESsT.

### 2.4. Cyclic Strain Causes Strong Induction of Osteogenic Marker Gene Expression in ESsT-C but Not in CTG-F Cells

**Figure 4:**
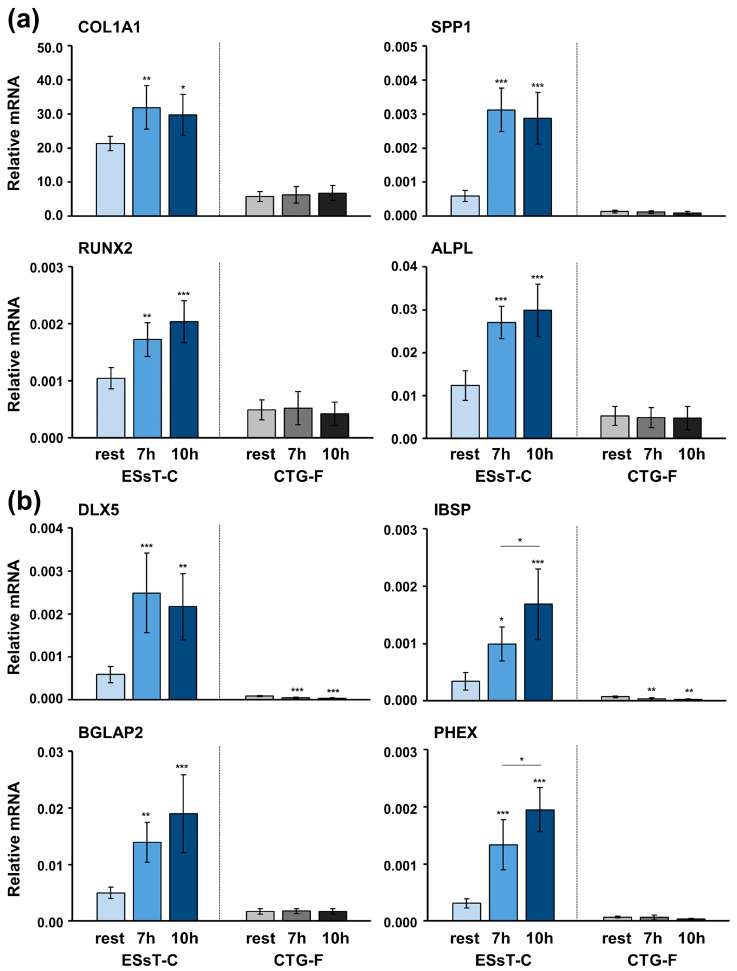
Cyclic strain causes strong induction of osteogenic marker gene expression in ESsT-C but not in CTG-F cells. Cyclic strain was applied to primary ESsT-Cs and CTG-Fs, as presented in Figure 3a. Total RNA was extracted from pelleted cells and qRT-PCR was performed for analyzing (**a**) COL1A1, SPP1, RUNX2, and ALPL and (**b**) DLX5, IBSP, BGLAP2, and PHEX transcripts normalized to GAPDH. Data represent means ± SD from six independent experiments performed with primary ESsT-Cs and CTG-Fs from six different donors. Significant differences to the respective resting control unless otherwise indicated, *** *p* < 0.001, ** *p* < 0.01, * *p* < 0.05 are shown.

Next, the effect of intermittent equibiaxial cyclic strain on the osteogenic marker gene expression in primary ESsT-Cs and CTG-Fs was investigated (Figure 4). Interestingly, primary ESsT-Cs exhibited preserved osteogenic potential both under basal conditions (rest) and under mechanical stimulation (7- and 10-h loading cycles) versus no osteogenesis in primary CTG-Fs under both conditions. Moreover, cyclic strain (both regimens) significantly upregulated the expression of both pre-osteoblast (Figure 4a) and osteoblast (Figure 4b) differentiation markers. A gradual increase, dependent on the duration of the cyclic strain application, was detected for most of the transcripts, including RUNX2, ALPL, IBSP, BGLAP2, and PHEX (Figure 4a,b). The strain-induced upregulation of two of the mature osteoblast markers, namely IBSP and PHEX, was significantly better pronounced with the 10-h compared to the 7-h regimen (Figure 4b). In contrast, in primary CTG-Fs, cyclic mechanical strain had no influence on the osteogenic gene expression and, interestingly, it caused a clear suppression of DLX5 and IBSP transcripts compared to resting cells.

### 2.5. Matrix Stiffness Triggers TGF-β1 and IGF-1 Expression in ESsT-Cs and CTG-Fs Thus Reflecting the Differences in the Growth Factor Gene Expression Observed at a Tissue Level

Our results demonstrated that ESsT-Cs and CTG-Fs had similar expression levels of IGF-1 and cyclic strain upregulated IGF-1 levels to the same extent (cf. Figure 3b,c). However, the question of whether the observed osteogenic potential of ESsT compared to CTG may be related to the differential expression levels of TGF-β1 in cells of the two tissue types remains unanswered. To investigate further the ESsT-Cs and CTG-Fs in a physiologically relevant environment close to their natural state in the oral tissues, we reproduced tissue stiffening in vitro. This approach was supported by the detected high expression of genes encoding ECM proteins, such as collagen type I and type III, periostin and osteopontin in the ESsT compared to the CTG, and the general enrichment of both tissues in ECM molecules (cf. Figure 1).

**Figure 5:**
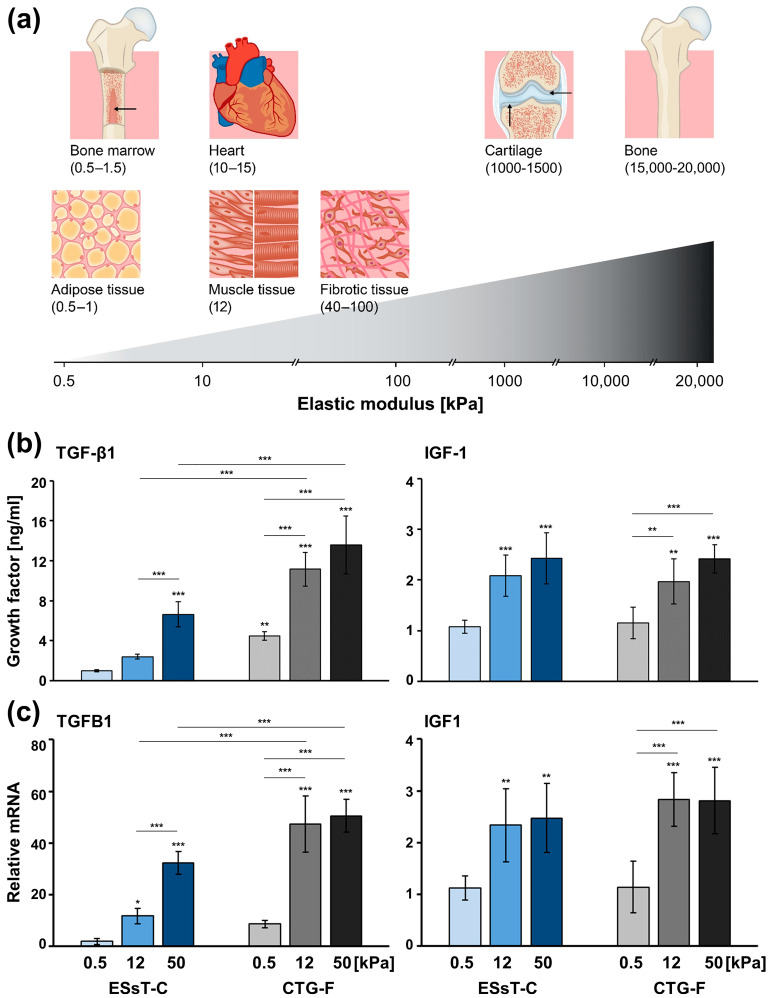
Matrix stiffness triggers TGF-β1 and IGF-1 expression in ESsT-Cs and CTG-Fs, thus reflecting the differences in the growth factor gene expression observed at a tissue level. (**a**) Schematic representation of native tissues and organs with their corresponding elastic moduli. (**b**) Analyses of TGF-β1 and IGF-1 protein content in culture supernatants of ESsT-Cs and CTG-Fs cultured on fibronectin-coated polyacrylamide hydrogels with a stiffness corresponding to either 0.5 (compliant), 12 (stiff), or 50 (very stiff) kPa elastic modulus. Values normalized to DNA content, to compensate for potential differences in the cell proliferation rate, are expressed relative to the values of control ESsT-Cs grown on 0.5 kPa matrices. Data represent means ± SD from six independent experiments performed with primary ESsT-C and CTG-F cells from six different donors. Significant differences to the ESsT-C on 0.5 kPa matrices unless otherwise indicated, *** *p* < 0.001, ** *p* < 0.01, * *p* < 0.05 are shown. (**c**) qRT-PCR analyses of TGFB1 and IGF1 mRNA levels. Values normalized to GAPDH are expressed relative to the values of ESsT-Cs grown on 0.5 kPa matrices. Data and statistical significance are presented as in (**b**).

ESsT-Cs and CTG-Fs were cultured on fibronectin-coated polyacrylamide hydrogels of defined stiffness, corresponding to 0.5, 12, or 50 kPa elastic modulus. This corresponds to the average stiffness measured in compliant tissues, such as adipose tissue or bone marrow (0.5 kPa), and more rigid tissues, such as muscle (12 kPa) or fibrotic tissue (~50 kPa), as it has been reported in the literature [15,32,33] and depicted in Figure 5a. It has to be noted that the chosen stiffnesses represent soft, intermediate, and high rigid conditions without pretending to mimic the stiffness of tissues surrounding the extraction socket. Similar to the effect of cyclic strain, the above-indicated stiffnesses exhibited no measurable effect on the cellular viability and proliferation (Supplementary Material, Figure S4). However, growing the cells on the stiff matrices (12 and 50 kPa) increased the cell spreading and stress fiber formation, as demonstrated by the F-actin stain shown in the Supplementary Material (Figure S5).

The TGF-β1 protein secreted by ESsT-Cs grown on soft 0.5 kPa matrices measured 1.01 ± 0.13 ng/mL versus 4.47 ± 0.41 ng/mL released by CTG-Fs cultured at the same stiffness (Figure 5b). Similar to the effect of cyclic strain, the stiffness sensed by ESsT-Cs grown on 12 kPa matrices caused a moderate upregulation of TGF-β1 protein levels not exceeding 2.42 ± 0.24 ng/mL and, thus, appeared not significantly different from the ESsT-Cs cultured on 0.5 kPa matrices. In contrast, the stiffness sensed by ESsT-Cs grown on the very rigid 50 kPa matrices caused a dramatic upregulation of TGF-β1 protein released in the culture supernatant by 6.6-fold, thus exceeding the average value of 4.6 ng/mL of TGF-β1 released by resting CTG-Fs or CTG-Fs cultured on soft matrices. Furthermore, the substrate rigidity was differently sensed by the CTG-Fs. Both rigid matrices, 12 and 50 kPa caused a gradual 2.5–3.0-fold increase in the TGF-β1 protein released by the CTG-Fs, reaching the high value of 13.59 ± 2.89 ng/mL on 50 kPa matrices. The TGF-β1 upregulation characteristic of CTG-Fs grown on both 12 and 50 kPa matrices appeared significantly different from the soft matrix controls as well as from the ESsT-Cs grown on the rigid matrices.

Similar to the cyclic strain application and in contrast to the TGF-β1, the quantity of IGF-1 protein secreted by ESsT-Cs and CTG-Fs grown on 0.5 kPa matrices did not differ and amounted to 1.08 ± 0.13 and 1.16 ± 0.31 ng/mL, respectively (Figure 5b). However, the 12 and 50 kPa rigidity of the matrices gradually increased the IGF-1 concentration by 1.7–2.3-fold for both cell types compared to the respective controls on 0.5 kPa matrices. Similar to the strain application, only the TGF-β1 release from CTG-Fs remained significantly higher than the release from ESsT-Cs under all three conditions whereas the IGF-1 release, despite being upregulated in cells grown on the rigid substrates, remained unchanged between the two cell types under all tested conditions.

Finally, the analyses of TGFB1 and IGF1 mRNA levels in ESsT-C and CTG-F cells cultured on 0.5, 12, and 50 kPa matrices fully reflected the results obtained at a protein level (Figure 5c).

Taken together, these results demonstrate that substrate stiffness, similarly to tensile stress is able to upregulate TGF-β1 and IGF-1 mRNA and protein levels while preserving the trend of expression of the two growth factors observed at a tissue level.

### 2.6. Induction of Osteogenic Marker Gene Expression in ESsT-Cs Strongly Depends on the Matrix Stiffness

**Figure 6:**
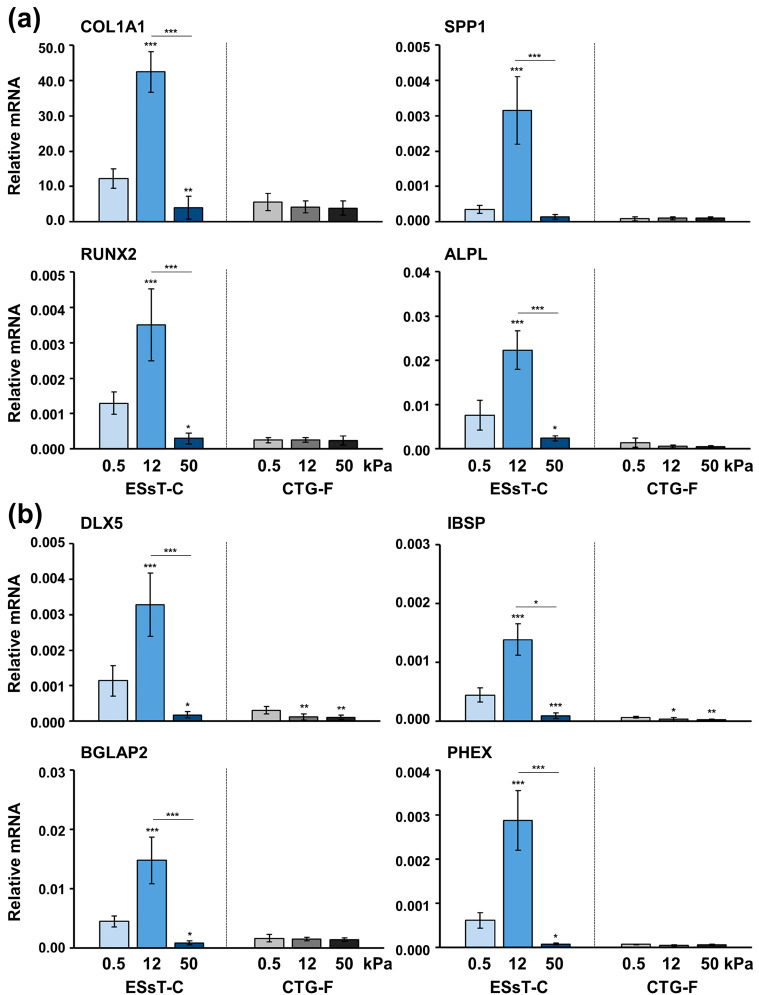
Induction of osteogenic marker gene expression in ESsT-Cs strongly depends on the matrix stiffness. Total RNA was extracted from primary ESsT-Cs and CTG-Fs cultured on fibronectin-coated polyacrylamide hydrogels with a stiffness corresponding to either 0.5 (compliant), 12 (stiff), or 50 (very stiff) kPa elastic modulus (cf. Figure 5a) and qRT-PCR was performed for analyzing (**a**) COL1A1, SPP1, RUNX2, and ALPL and (**b**) DLX5, IBSP, BGLAP2, and PHEX transcripts normalized to GAPDH. Data represent means ± SD from six independent experiments performed with primary ESsT-C and CTG-F cells from six different donors. Significant differences to the respective control lines grown on 0.5 kPa matrices unless otherwise indicated, *** *p* < 0.001, ** *p* < 0.01, * *p* < 0.05 are shown.

Next, osteogenic marker gene expression was investigated in primary ESsT-Cs and CTG-Fs grown on matrices with different stiffnesses (Figure 6). The primary ESsT-Cs grown on 0.5 and 12 kPa matrices exhibited preserved osteogenic potential versus no osteogenesis detected in primary CTG-Fs under both conditions. Both pre-osteoblast (Figure 6a) and osteoblast (Figure 6b) differentiation markers were extremely significantly upregulated by 2.7–9.0–fold in ESsT-Cs grown on 12 compared to the cells grown on 0.5 kPa matrices. Interestingly, all osteoblast differentiation markers, except for SPP1, were significantly (*p* < 0.05) downregulated by 3.1–8.5-fold in ESsT-Cs cultured on 50 kPa compared to 0.5 kPa matrices. Similar to the effect of cyclic mechanical strain, the matrix stiffness in primary CTG-Fs had no influence on the osteogenic gene expression. Moreover, in most cases, a gradual inhibition, significant in the case of DLX5 and IBSP mRNA levels, was observed in the CTG-Fs grown on 12 and 50 kPa matrices compared to cells grown on compliant matrices (Figure 6b).

Taken together, our results strongly suggest that variations in the concentration of TGF-β1, likely in a combination with a steady-state level of IGF-1, strongly influence the osteogenic differentiation of ESsT-Cs originating from the extraction socket at 8 weeks of healing.

### 2.7. Local Concentrations of TGF-β1 and IGF-1 Appear Determinant for the Osteogenic and Mineralization Potential of Primary ESsT-Cs

**Figure 7:**
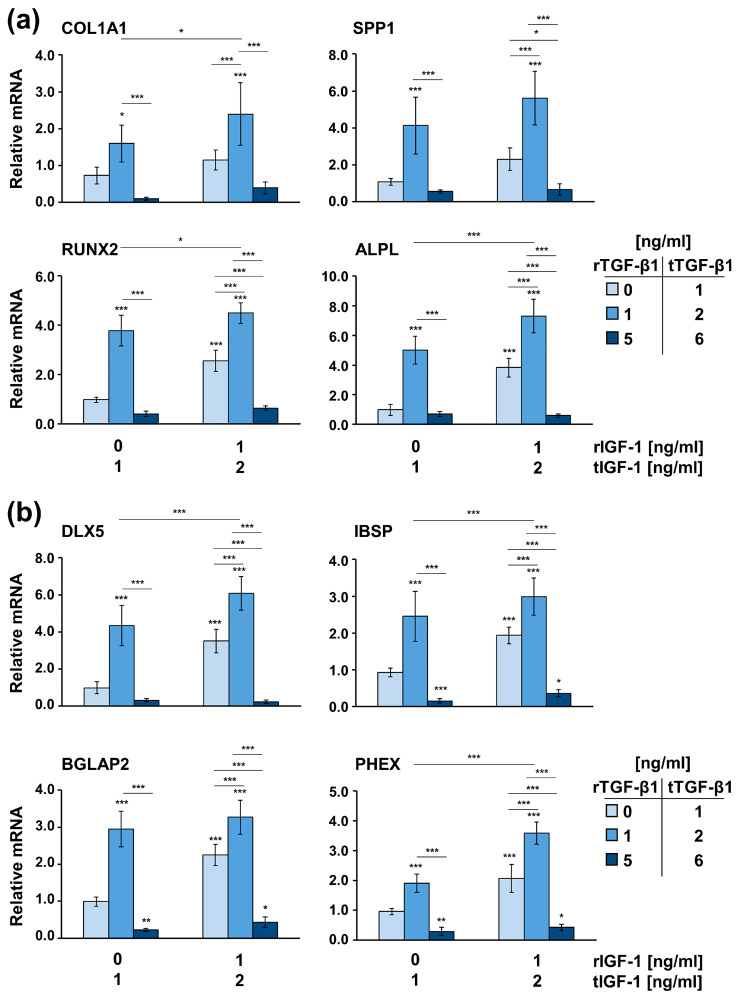
Local concentrations of TGF-β1 and IGF-1 appear determinant for the osteogenic potential of primary ESsT-Cs. Cells were treated with increasing concentrations (0, 1, or 5 ng/mL) of recombinant (r) TGF-β1 in the absence (0 ng/mL) or presence (1 ng/mL) of recombinant (r) IGF-1 for 24 h before total RNA was extracted. Please note that primary ESsT-Cs produce and secrete endogenous levels of TGF-β1 and IGF-1 to the average value of 1 ng/mL for each of the two growth factors (cf. Figure 3 and Figure 5). Therefore, the total (t) concentration of each of the two growth factors, summing the endogenous and recombinant concentrations, is indicated. Expression of (**a**) COL1A1, SPP1, RUNX2, and ALPL and (**b**) DLX5, IBSP, BGLAP2, and PHEX mRNAs was analyzed by qRT-PCR. Values normalized to GAPDH are expressed relative to the values of untreated cells. Means ± SD from six independent experiments performed with primary ESsT-Cs from six different donors, and significant differences to untreated cells unless otherwise indicated, *** *p* < 0.001, ** *p* < 0.01, * *p* < 0.05 are shown.

Finally, to investigate how exogenously applied, recombinant (r) TGF-β1 and IGF-1 influence the osteogenic and mineralization capacity of primary ESsT-Cs, the cells were treated with increasing concentrations (0, 1, or 5 ng/mL) of rTGF-β1 in the absence (0 ng/mL) or presence (1 ng/mL) of rIGF-1 (Figure 7). Since the endogenous levels of TGF-β1 and IGF-1 produced and secreted by the ESsT-Cs amounted to the average value of 1 ng/mL for each of the two growth factors (cf. Figure 3 and Figure 5), the detection of a pure paracrine activity of the growth factors was not possible. For the sake of clarity, the total (t) concentration of each of the two growth factors, summing the endogenous and recombinant concentrations, is indicated in the figure.

Our data showed that 1 ng/mL of rTGF-β1 (equal to 2 ng/mL of tTGF-β1) applied either in the absence of rIGF-1 (equal to 1 ng/mL of tIGF-1) or the presence of 1 ng/mL of rIGF-1 (equal to 2 ng/mL of tIGF-1) caused a significant increase in COL1A1, SPP1, RUNX2, ALPL, DLX5, IBSP, BGLAP2, and PHEX mRNAs compared to the respective expression levels in cells not receiving recombinant growth factors exogenously, from here on referred to as untreated cells (Figure 7a,b). Similar to the effect seen in ESsT-Cs grown on stiff 50 kPa matrices (cf. Figure 6), 5 ng/mL of rTGF-β1 (equal to 6 ng/mL of tTGF-β1) applied in the presence of IGF-1 as indicated above caused a strong suppression of the osteogenic gene expression. In the case of late differentiation markers such as IBSP, BGLAP2, and PHEX, the suppression appeared significant compared to their expression levels in untreated cells (Figure 7b). Except for the expression of genes encoding the bone matrix proteins collagen type 1 and osteopontin (Figure 7a), the application of 1 ng/mL of rIGF-1 (equal to 2 ng/mL of tIGF-1) in the absence of rTGF-β1 (equal to 1 ng/mL of tTGF-β1) caused an extremely significant upregulation of all osteoblast-specific mRNAs above the expression levels seen in untreated cells (Figure 7). Furthermore, in the presence of 2 ng/mL of tIGF-1, 2 ng/mL of tTGF-β1 significantly potentiated the osteogenic gene expression compared to 1 ng/mL of tTGF-β1, which supported the dose-dependent and synergistic activity of TGF-β1. In the presence of 2 ng/mL of tTGF-β1, except for SPP1 and BGLAP2 mRNAs, all other osteogenic transcripts were significantly better induced by 2 ng/mL of tIGF-1 compared to 1 ng/mL of tIGF-1, which further supported a mutual synergism between the two growth factors in triggering the osteogenic potential of ESsT-Cs.

**Figure 8:**
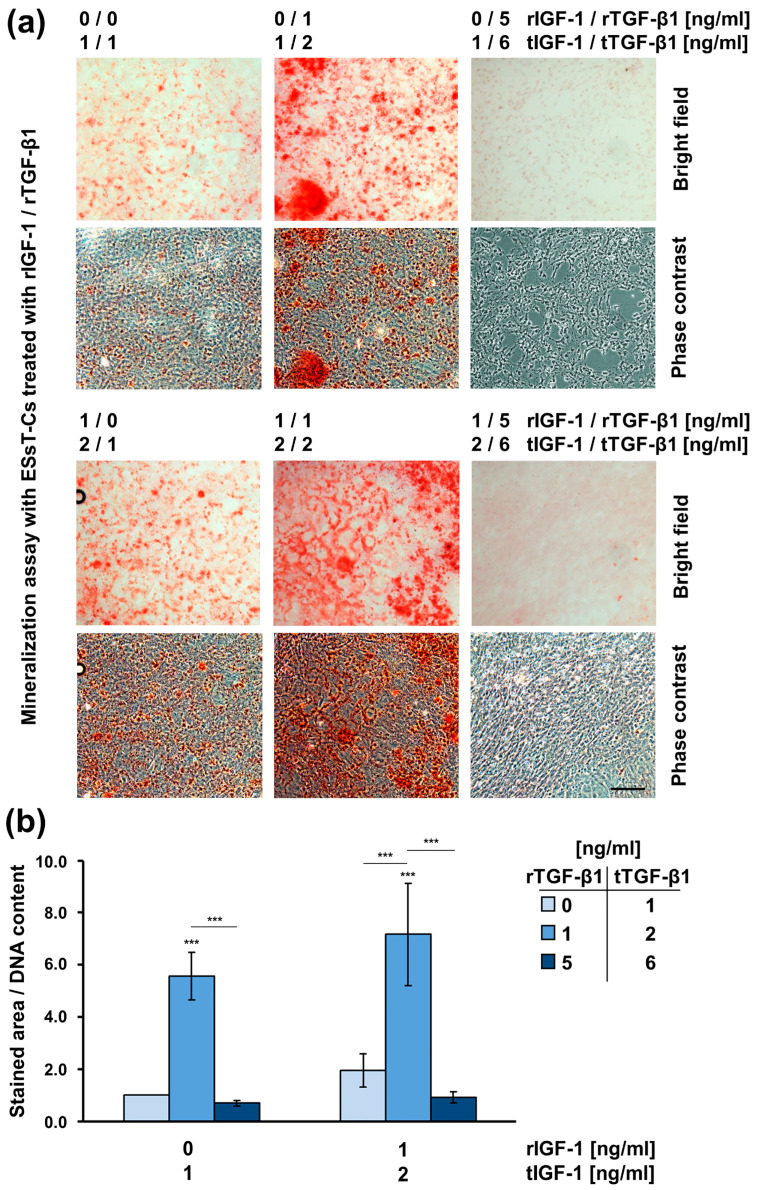
Local concentrations of TGF-β1 and IGF-1 appear determinant for the mineralization potential of primary ESsT-Cs. (**a**) Cells were treated with increasing concentrations (0, 1, or 5 ng/mL) of recombinant (r) TGF-β1 in the absence (0 ng/mL) or presence (1 ng/mL) of recombinant (r) IGF-1 for 4 days and subsequently grown under osteogenic culture conditions for 10 additional days before extracellular matrix mineralization was assessed by alizarin red stain. For clarity, the total (t) concentrations of each of the two growth factors, summing the endogenous and recombinant concentrations, are also indicated. Representative bright field and phase contrast images are shown. Scale bar, 500 µm. (**b**) Mineral deposition capacity was quantified by measuring the stained area using the Fiji distribution of ImageJ. Values normalized to DNA content are expressed relative to the values of untreated cells. Means ± SD from three independent experiments performed with primary ESsT-Cs from three different donors and significant differences to untreated cells, unless otherwise indicated, *** *p* < 0.001 are shown.

Next, we assessed the mineralization capacity of untreated but containing endogenous growth factors versus recombinant growth factor-treated ESsT-Cs (Figure 8). Our data have shown that, compared to untreated cells, mineralization was slightly but not significantly stimulated by the treatment of the cells with rIGF-1 only. ESsT-Cs exposed to concentrations of tTGF-β1 not exceeding 2 ng/mL, in the presence of either 1 or 2 ng/mL tIGF-1, showed a dramatic increase by 5.6- or 7.2-fold, respectively, in their mineralization capacity compared to untreated cells. Finally, ESsT-Cs exposed to concentrations of tTGF-β1 of 6 ng/mL, in the presence of either 1 or 2 ng/mL tIGF-1, exhibited no mineralization potential.

Altogether, our results strongly suggest that the local concentrations of TGF-β1 and IGF-1 growth factors in the postextraction tooth socket at 8 weeks of healing potentiate the differentiation of ESsT-Cs toward the osteoblast lineage.

## 3. Discussion

The present study aimed at characterizing human tissue samples from the postextraction tooth socket at 8 weeks of healing, representing repaired tissue that undergoes remodeling, and comparing them with palatal subepithelial CTGs, representing healthy, never-healed tissue that is commonly used for soft tissue augmentations in dental implant surgery. We hypothesized that the activation of certain molecular cues by the normal sequence of events during the healing process might grant the repaired postextraction tissue a regenerative advantage. Furthermore, we aimed to clarify whether specific molecular signals in the postextraction soft tissue would classify it for use in soft or hard tissue regeneration and augmentation procedures. Despite not being able to answer these questions in full, our data shed significant light on the similarities and differences between the two tissue types and allowed us to draw important conclusions: (a) the ESsT and CTG share characteristics of nonspecialized soft connective tissue by expressing comparable levels of genes encoding abundant ECM proteins; (b) the ESsT shows clear characteristics of specialized hard connective tissue by expressing high levels of osteogenic differentiation markers; and (c) TGF-β1 and its two receptors are strongly enriched in the CTG whereas IGF-1 shows significantly high and comparable expression in both ESsT and CTG. Finally, in a series of in vitro experiments simulating a tissue-relevant physiological environment, namely mechanical stimulation of oral tissues, we were able to show that local concentrations of TGF-β1 and IGF-1 appear determinant in regulating bone regeneration in the postextraction tooth socket.

The IGF-1 growth factor has a well-known osteogenic activity [34] and its levels in the circulation have been positively associated with bone mass in humans [35,36]. A positive effect of systemic administration of rIGF-1 on alveolar bone formation in healthy and diabetic rats has also been reported [37,38]. Micro-computed tomography analysis has shown an increase in newly formed alveolar bone during and after a 3-week subcutaneous administration of 320 mg/day of rIGF-1 to healthy rats that had avulsion of the right mandibular first molar. In addition, a significant reduction in the alveolar ridge height loss after dental avulsion was also observed [38]. Despite our finding of significant IGF1 mRNA expression, no significant differences were observed between ESsT and CTG tissues. Thus, the osteogenic potential of the ESsT cannot be solely attributed to the IGF-1.

In contrast, TGF-β1 is known to promote the recruitment of osteoprogenitors, their proliferation, and bone matrix production [39,40]. However, TGF-β1 exhibits an inhibitory effect on osteoblast differentiation and maturation [41,42] and the mechanisms of this negative regulation are not well understood. Furthermore, the TGF-β1 plays a pivotal role in the ECM synthesis throughout the soft tissue healing process [43]. Both TGFβR1 and TGFβR2 bind each of the three isoforms of TGF-β with different affinities, higher for TGF-β1 [44]. Due to the significant expression of the two receptors in the CTG and the autocrine as well as paracrine activity of the TGF-β growth factors [45], it is to be expected that the cells composing the CTG are not only producing high TGF-β1 levels but will also respond to all three TGF-β isoforms produced and secreted by either themselves or cells of nearby tissues that migrate to the soft tissue defect covered by the CTG. Thus, our data support the use of the CTG primarily for soft tissue grafting.

To investigate a potential interplay between the two growth factors, we were prompted to look for an in vitro setup that mimics the physiological environment and will thus reproduce the differences in the growth factor gene expression observed at a tissue level. Oral soft tissues are constantly subjected to a wide variety of mechanical forces, including hydrodynamic forces, strain, compression, friction, and shear generated during saliva flow, chewing, speaking, and tooth brushing [14,15]. Based on studies that have quantified the magnitude and the frequency corresponding to mastication studied in laboratory [46,47,48] and natural environments [49], we have designed two loading regimens alternating 1-h strain (10%, 1 Hz) with 2-h resting intervals for the total duration of 7 or 10 h. In this way, we replicated an average, in magnitude and frequency, mechanical irritation corresponding to a short- or long-duration mechanical loading of all different types (as listed above) within a day. It should be noted that the magnitude and frequency of mechanical stimulation greatly depend on the location of the tissue [14,47], the diet and lifestyle of the subjects, including the physical properties of the food [50], food volume [51], hardness, and texture [52]. Along with mechanical forces applied to them, cells can also sense the stiffness of their microenvironment. It has been demonstrated that inhibition of cell spreading due to the lack of matrix stiffness can be overcome by externally applied cyclic strain, suggesting that similar mechanotransduction mechanisms sense stiffness and strain [53]. Our data have shown that several collagen transcripts including COL1A1, COL1A2, and COL3A1 as well as other ECM molecules such as POSTN and SPP1 are enriched in the ESsT, which in itself may increase the stiffness that cells composing this tissue type experience from the surrounding matrix. Type I collagen deposition has been shown to correlate linearly with tissue stiffness in many different organs [37,38]. In fact, matrix stiffening has two main causes: (1) stiffening due to increased deposition of ECM molecules, mostly collagens, and (2) stiffening due to strain [54]. Furthermore, it has been shown that mechanical stimulation increases the expression of both TGF-β1 [55] and IGF-1 [56]. Moreover, both growth factors are able to sense mechanical forces: TGF-β1 is involved in mechanosensing pathways through the activation of β6, αV, and β8 integrins [57] whereas IGF-1 through the formation of an IGF1R/αVβ3 integrin complex [58]. In this regard, the approach of examining the response of ESsT-C and CTG-F cells to mechanical stimulation by sensing strain or stiffness was a logical step in our study that proved to reflect the changes in gene expression observed at a tissue level.

Both types of mechanical stimulation applied to primary ESsT-Cs revealed that cyclic strain- or stiffness-induced TGF-β1 expression not exceeding an average value of 2.3 ng/mL in combination with IGF-1 expression up to an average of 2.5 ng/mL was able to induce the osteogenic potential of ESsT-Cs. Culturing of the ESsT-Cs on very rigid (50 kPa) matrices, upregulating the TGF-β1 to the average value of 6.6 ng/mL, caused a strong downregulation of osteogenic gene expression. However, whether TGF-β1 is a direct or indirect mediator of the effects caused by the mechanical stimulation of primary ESsT-Cs needs to be further verified in experiments utilizing chemical or biological inhibition of the growth factor. In primary CTG-Fs, endogenous or stress-induced TGF-β1 that was equal to or greater than the average value of 4.6 ng/mL, respectively, likely suppressed the osteogenic activity of IGF-1 and might explain the complete lack of osteogenesis in CTG-Fs. This was proven further in a setup where the primary ESsT-Cs were treated with exogenously applied growth factors as if the cells were under the simultaneous influence of autocrine and paracrine acting growth factors delivered by cells migrating in the healing extraction socket. This physiologically relevant situation proved a mutual synergistic effect of the two growth factors in inducing the osteogenic potential of the ESsT-Cs and validated further that namely the specific local concentrations of the two growth factors determine the hard tissue characteristics of the ESsT. In particular, TGF-β1 concentrations not exceeding 2 ng/mL in combination with comparable concentrations of IGF-1 had synergistic effects in inducing osteogenic gene expression in the primary ESsT-Cs. However, TGF-β1 concentrations equal to 6 ng/mL in combination with IGF-1 not exceeding 1 ng/mL or slightly increased up to 2 ng/mL suppressed the osteogenic as well as the mineralization capacity of the ESsT-Cs. It has to be noted that our experimental setup did not investigate TGF-β1 concentrations in the range of 2–6 ng/mL as well as IGF-1 concentrations higher than 2 ng/mL. Our studies extend and corroborate those of previous studies using different model systems, indicating that the combination of IGF-1 with other growth factors, such as PDGF, FGF-2, or TGF-β1, triggers synergistic responses at both cellular and tissue levels and promotes bone formation above that found with single growth factors [59,60,61,62,63,64]. In line with our findings, studies have also reported biphasic and concentration-dependent effects of TGF-β1 on osteoblast differentiation [65,66]. Ochiai et al. have shown that repeated administration of rTGF-β1 to cultures of human periodontal ligament cells, human mesenchymal stem cells, or murine preosteoblast MC3T3-E1 cells inhibited their osteogenic differentiation by causing a remarkable decrease in the mRNA and protein expression levels of the IGF-1 [66]. Previous evidence has also suggested that each of the three growth factors TGF-β1, PDGF-BB, and FGF-2 were able to downregulate IGF-1 transcript and protein levels in bone cells [67]. In contrast, our data have shown that both strain- and stiffness-induced TGF-β1 expression in each of the two primary cell lines ESsT-C and CTG-F caused no downregulation of IGF1 mRNA and IGF-1 protein levels. In contrast, the TGF-β1 induction caused by the mechanical stimulation of the primary cells coincided with a significant increase in the IGF-1 expression above the levels detected in control cells. Differing cell origin and culture conditions are likely responsible for some of the differences observed in our study. The current investigation appears unique in (1) focusing specifically on primary cells extracted from the ESsT at 8 weeks of healing, a frequently used time point for implant placement, and their comparison with primary cells extracted from palatal CTG, and (2) applying physiologically relevant cell culture conditions reproducing mechanical stimulation in the oral cavity. The fact that the hormetic-like biphasic cellular response to changing concentrations of TGF-β1/IGF-1 was observed within the context of different experimental setups, namely upon culturing ESsT-Cs in rigid matrices as well as their treatment with recombinant growth factors, is an important observation that requires a better understanding of the mechanistic basis of growth factor-induced hormetic dose responses. Further studies investigating the downstream signaling pathways of TGF-β1 and IGF-1 in the ESsT-Cs and CTG-Fs are warranted for (1) better verification of these initial findings, (2) exploring the concept of hormesis [68,69] in the context of postextraction socket healing, and (3) exploring the ways for the enhancement of both soft and hard tissue regeneration.

From a clinical perspective, our study suggests that, instead of being discarded, the ESsT at 8 weeks of healing can be left attached to the mucoperiosteal flap and be placed in proximity to osseous defects or sites undergoing hard tissue augmentations, since, being highly enriched in osteogenesis-related markers, it may create a more mature bone healing environment. To our knowledge, extraction socket tissue has so far been utilized and investigated as a graft material only once in a dog study [70]. However, the extraction sockets did not heal spontaneously but were instead treated with a methylcellulose gel containing PDGF-BB and IGF-1 at concentrations of 6 µg/mL each over 5 days before their use as graft material in Class II furcation defects created in the second, third, and fourth lower premolars. In this clinical scenario, no significant difference was observed between the test and control groups in terms of the formation of connective tissue, new cementum, new bone, and junctional epithelium [70].

Furthermore, the observed high enrichment of osteogenic differentiation markers in the ESsT at 8 weeks of healing excludes its potential application as a soft tissue substitute and alternative of the subepithelial palatal CTG. Moreover, our data suggest that a CTG should not be applied in a close proximity to osseous defects, where the high TGF-β1 content of the CTG can potentially inhibit the osteogenic activity of locally present IGF-1. Whereas several publications report the combinatorial application of IGF-1 with PDGF or VEGF (summarized in [71]), only two preclinical studies have evaluated the application of rhIGF-1 together with rhTGF-β1 [72,73]. The effect of titanium membranes coated with rhIGF-1 and rhTGF-β1 (1% w/w) in the regeneration of transosseous defects created in the mandibular rami of Spargue–Dawley rats was investigated at 28 days of healing [72]. The authors concluded that, despite acceleration of the healing process of the bony defects, the combination of the two growth factors did not improve the bone quality. In another study, a hydrogel scaffold loaded with 25 ng of IGF-1 and 0.1 µg of TGF-β1 was implanted in rat mandibular bone defects that were allowed to heal for 3 and 6 weeks before radiological and morphological analyses were performed [73]. The closure of the defects was significantly accelerated in the presence of the growth factors compared to the control groups treated with either saline alone or saline-containing hydrogel. However, there was no significant advantage in the healing of the defects with the combination of the growth factors versus their individual application, which, as suggested by our study, is most probably due to the combinatorial effect of the selected concentrations for the two growth factors.

Our previous investigations have shown that particulate autogenous bone releases TGF-β1 in a very short period of only 10 min [40]. Bone-conditioned medium (BCM) prepared from autologous bone particles and used for functionalization of biomaterials, e.g., bone substitute and/or a collagen barrier membrane, during a GBR procedure appears highly enriched in physiologically effective amounts of TGF-β1 [40,74]. Potentially, in the presence of TGF-β1 absorbed on the barrier membrane, an ESsT graft would not manifest its osteogenic potential. This raises the possibility that, if an ESsT is not removed from the mucoperiosteal flap, it can serve as a second barrier along with a BCM-coated collagen membrane used in the GBR procedure. This will not only stabilize the flap, but may prove beneficial to the soft tissue healing process around the implant site, as the ESsT and CTG appear to be equally rich in ECM molecules. Future in vitro and in vivo research is needed to reveal the full regenerative potential of the ESsT. Its potential application as a graft must be explored clinically in periodontal and implant surgical therapies.

Despite the fact that the 8 weeks of healing is a frequently used scenario in early implant placement with GBR [75], a clear limitation of our in vitro study is the lack of comparison with ESsTs obtained at different time points post extraction. Future in vitro studies characterizing the tissue at more than one healing time point and on a larger scale, namely bigger sample size and, e.g., transcriptome analyses by next-generation sequencing, are needed.

Over the past decade, the wound healing therapeutic strategies have significantly focused on the action of various growth factors and suitable growth factor delivery systems [76]. Therefore, our findings on the interplay between TGF-β1 and IGF-1 as two growth factors with a significant impact on the healing process put us a step forward in our understanding whether and how growth factors for therapeutic use can or cannot be combined. Knowledge about the basic biological principles of healing can guide the oral surgeon in the selection of appropriate biologics to enhance bone or soft tissue regeneration.

## 4. Materials and Methods

### 4.1. Tissue Samples and Cell Culture

ESsT was biopsied from systemically and periodontally healthy subjects (in total 6; ≥18 years old) at the time of implant placement, namely 8 weeks post extraction. In parallel, a palatal CTG was harvested from the same patient. The Ethics Committee, Bern, Switzerland (ethical code ID 2018-00661 from 13 August 2018) approved the study and informed consent was obtained from all patients. Immediately after the excision, each tissue sample was divided into two pieces. One of them was immediately placed in RNAlater (ThermoFisher Scientific, Basel, Switzerland) for 20 h before tissue homogenization using gentleMACS Dissociator (Miltenyi Biotec, Bergisch Gladbach, Germany), followed by RNA extraction using the RNeasy Fibrous Tissue Mini Kit (Qiagen, Basel, Switzerland). The second tissue piece was placed in cell culture medium and used for extraction of primary ESsT-C and CTG-F cells by the tissue explant technique as described [77]. Primary cells derived from 1 mm-sized tissue explant pieces were cultured in Dulbecco’s Modified Eagle Medium (DMEM; ThermoFisher Scientific) supplemented with 10% fetal calf serum (FCS; ThermoFisher Scientific) and 1% antimycotics/antibiotics (ThermoFisher Scientific). Primary cells that had not undergone more than four passages were starved in 0.3% FCS/DMEM before culturing under experimental conditions.

In some cases, the cells were treated with recombinant TGF-β1 and/or IGF-1 proteins (PeproTech, London, UK) for 24 h (RNA analyses) or 4 days (mineralization analyses). For mineralization analyses, media were supplemented with 50 μg/mL ascorbic acid (Invitrogen, ThermoFisher Scientific) and 2 mM β-glycerophosphate (Invitrogen, ThermoFisher Scientific) as described [40].

### 4.2. Mechanical Stimulation of Cells by Using Flexcell^®^ Tension System and Matrix Stiffening

A total of 2 × 10^5^ ESsT-C or CTG-F cells/well were seeded on fibronectin-coated silicone membranes in BioFlex^®^ 6-well culture plates (Flexcell International, Hillsborough, NC, USA) as described [78,79]. Cultures were starved for 24 h before applying intermittent equibiaxial cyclic strain (10%, 1 Hz) at 37 °C with the loading cycles described in Figure 3a using the Flexcell FX-4000 computer-controlled vacuum system (Flexcell International). Cells cultured under the same conditions and not exposed to strain were used as a resting control.

For matrix stiffness experiments, 2 × 10^5^ ESsT-C or CTG-F cells/well were seeded in 0.3% FCS/DMEM on fibronectin-coated polyacrylamide hydrogels of defined stiffness (0.5, 12, or 50 kPa elastic modulus) in 35 mm Petrisoft^TM^ dishes (Matrigen, Irvine, CA, USA) for 16 h as described [80].

After mechanical stimulation, cell culture supernatants were analyzed by ELISA whereas cells were either fixed for phalloidin stain or lysed for RNA extraction using the RNeasy Mini Kit (Qiagen). In some cases, cell viability and proliferation were assessed by the CellTiter-Blue cell viability assay (Promega, Dübendorf, Switzerland) and the BrdU Cell Proliferation ELISA (Roche, Basel, Switzerland), respectively.

### 4.3. Phalloidin Stain

After being fixed in Formal-Fixx (ThermoFisher Scientific) for 10 min, cells were washed and blocked in phosphate-buffered solution (PBS) containing 3% bovine serum albumin (Sigma, Buchs, Switzerland) and 0.1% Triton X-100 (Sigma) for 10 min. Cells were then labeled with Alexa Fluor^TM^ 488 Phalloidin (Invitrogen, ThermoFisher Scientific) or Phalloidin-TRITC (Bio-Techne, Zug, Switzerland) at room temperature for 1 h. Cells were washed in PBS containing 0.1% Triton X-100 and co-stained with 4′,6-diamidino-2-phenylindole (DAPI; Sigma) at the last washing step before being mounted in Vectashield Medium (Adipogen, Fuellinsdorf, Switzerland). Images were acquired on an Olympus BX-51 (Olympus Life Sciences Solution, Tokyo, Japan) equipped with the fluorescent filters U-MWIBA3 for Alexa Fluor 488, U-MWIGA3 for TRITC, and U-MNUA2 for DAPI.

### 4.4. ELISA Protein Quantification

Release of TGF-β1 and IGF-1 proteins in cell culture supernatants was quantified using Quantikine^®^ colorimetric sandwich ELISA (R&D Systems, Zug, Switzerland) according to the manufacturer’s procedure. Absorbance was measured at 450 and 570 nm on an ELx808 Absorbance Reader (BioTek, Luzern, Switzerland). Data represent means ± SD for six independent experiments performed with the six different cell donors for the primary ESsT-Cs and CTG-Fs.

### 4.5. Gene Expression Analyses by qRT-PCR

qRT-PCR was used to investigate the expression of four groups of genes: (1) osteogenic markers (COL1A1, SPP1, RUNX2, ALPL, DLX5, IBSP, BGLAP2, and PHEX), (2) genes encoding ECM proteins (COL1A2, COL3A1, POSTN, FN1, VIM, and TNC), (3) genes encoding isoforms of FGF and BMP proteins and their receptors (FGF2, FGFR1, FGFR2, FGFR3, FGFR4, BMP2, BMP4, BMP7, BMPR1A, BMPR1B, BMPR2, and ACVR1; for details, see Supplementary Material), and (4) genes encoding different isoforms of TGF-β and IGF proteins and their receptors (TGFB1, TGFB2, TGFB3, TGFBR1, TGFBR2, IGF1, IGF2, IGFR1, and IGFR2).

Total RNA from ESsT and CTG tissue samples or primary ESsT-Cs and CTG-Fs was spectrophotometrically quantified on a NanoDrop 2000c instrument (ThermoFisher Scientific) and then reverse transcribed using the Applied Biosystems™ High-Capacity cDNA Reverse Transcription Kit (ThermoFisher Scientific) as described [81]. Relative transcripts for the above listed genes, normalized to GAPDH, were quantified using FastStart Universal SYBR Green Master ROX (Roche, Basel, Switzerland) and the primer sequences listed in Tables S1–S4. Quantitative PCR was carried out in a QuantStudio 3 instrument (Applied Biosystems, Rotkreuz, Switzerland) using a standard thermal cycling profile. Data were analyzed by the ∆Ct or ∆∆Ct method that included a calibration to values of controls. Data represent means ± SD from six independent experiments performed with the six different cell donors for the primary ESsT-Cs and CTG-Fs.

### 4.6. Alizarin Red Stain

The mineralization capacity of growth factor-treated ESsT-Cs was analyzed by alizarin red stain after 14 days using 0.2% Alizarin Red S (Sigma), pH 6.4 as described [40]. Images were acquired on an Olympus BX-51 (Olympus Life Sciences Solution). Mineral deposition was quantified by measuring the stained area using ImageJ as described [40]. Cellular DNA content, measured by the CyQUANT^®^ NF assay (Invitrogen, ThermoFisher Scientific), was used for normalization. Data represent means ± SD from three independent experiments performed with primary ESsT-Cs from three different donors.

### 4.7. Statistical Analysis

The statistical analyses were carried out using GraphPad InStat Software, version 3.05. Differences between two groups were assessed by Student’s *t*-test and between multiple groups by one-way analysis of variance (ANOVA) with Tukey’s post hoc test. Values of *p* < 0.05 were considered statistically significant.

## Data Availability

All data generated and analyzed during this study are included in this article.

## Supplementary Materials

The following supporting information can be downloaded at: https://www.mdpi.com/article/10.3390/ijms24098239/s1.
