# Supplementary material for: Local Concentrations of TGF-β1 and IGF-1 Appear Determinant in Regulating Bone Regeneration in Human Postextraction Tooth Sockets
Source: Int J Mol Sci. 2023 May 4;24(9):8239. doi: 10.3390/ijms24098239 (PMC10179638; doi:10.3390/ijms24098239)
Supplement: Supplementary file 1 [file ijms-24-08239-s001.zip › ijms-2341553-supplementary.pdf]

Supplementary Material for:

Article

Local concentrations of TGF-β1 and IGF-1 appear determinant in regulating bone regeneration in human postextraction tooth sockets

Maria B. Asparuhova [1,2,*], Dominic Riedwyl [1,2], Ryo Aizawa [1,2,3], Clemens Raabe [2], Emilio Couso-Queiruga [2] and Vivianne Chappuis [2]

[1] Laboratory of Oral Cell Biology, Dental Research Center, School of Dental Medicine, University of Bern, Freiburgstrasse 3, 3010 Bern, Switzerland; mariya.asparuhova@unibe.ch; dominic.riedwyl@unibe.ch
[2] Department of Oral Surgery and Stomatology, School of Dental Medicine, University of Bern, Freiburgstrasse 7, 3010 Bern, Switzerland; clemens.raabe@unibe.ch; emilio.couso@unibe.ch; vivianne.chappuis@unibe.ch
[3] Department of Periodontology, School of Dentistry, Showa University, 2-1-1 Kitasenzoku, Ohta-ku, Tokyo 145-8515, Japan; r-aizawa@dent.showa-u.ac.jp
* Correspondence: mariya.asparuhova@unibe.ch

Figure S1: No significant changes in the expression of genes encoding (a) FGF-2 and its receptors, and (b) BMP-2, -4, -7 and their receptors in ESsT and CTG samples.

Figure S2: No significant changes in cell viability and proliferation of primary ESsT-Cs and CTG-Fs subjected to intermittent equibiaxial cyclic strain.

Figure S3: Morphological appearance and actin stress fiber formation in primary ESsT-Cs and CTG-Fs subjected to intermittent equibiaxial cyclic strain.

Figure S4: No significant changes in cell viability and proliferation of primary ESsT-Cs and CTG-Fs grown on soft (0.5 kPa) or stiff (12 or 50 kPa) matrices.

Figure S5: Morphological appearance and actin stress fiber formation in primary ESsT-Cs and CTG-Fs grown on soft (0.5 kPa) or stiff (12 or 50 kPa) matrices.

Table S1: Primer sequences for osteogenic marker genes.

Table S2: Primer sequences for genes encoding extracellular matrix proteins.

Table S3: Primer sequences encoding isoforms of FGF and BMP and their receptors.

Table S4: Primer sequences encoding isoforms of TGF-β and IGF and their receptors.

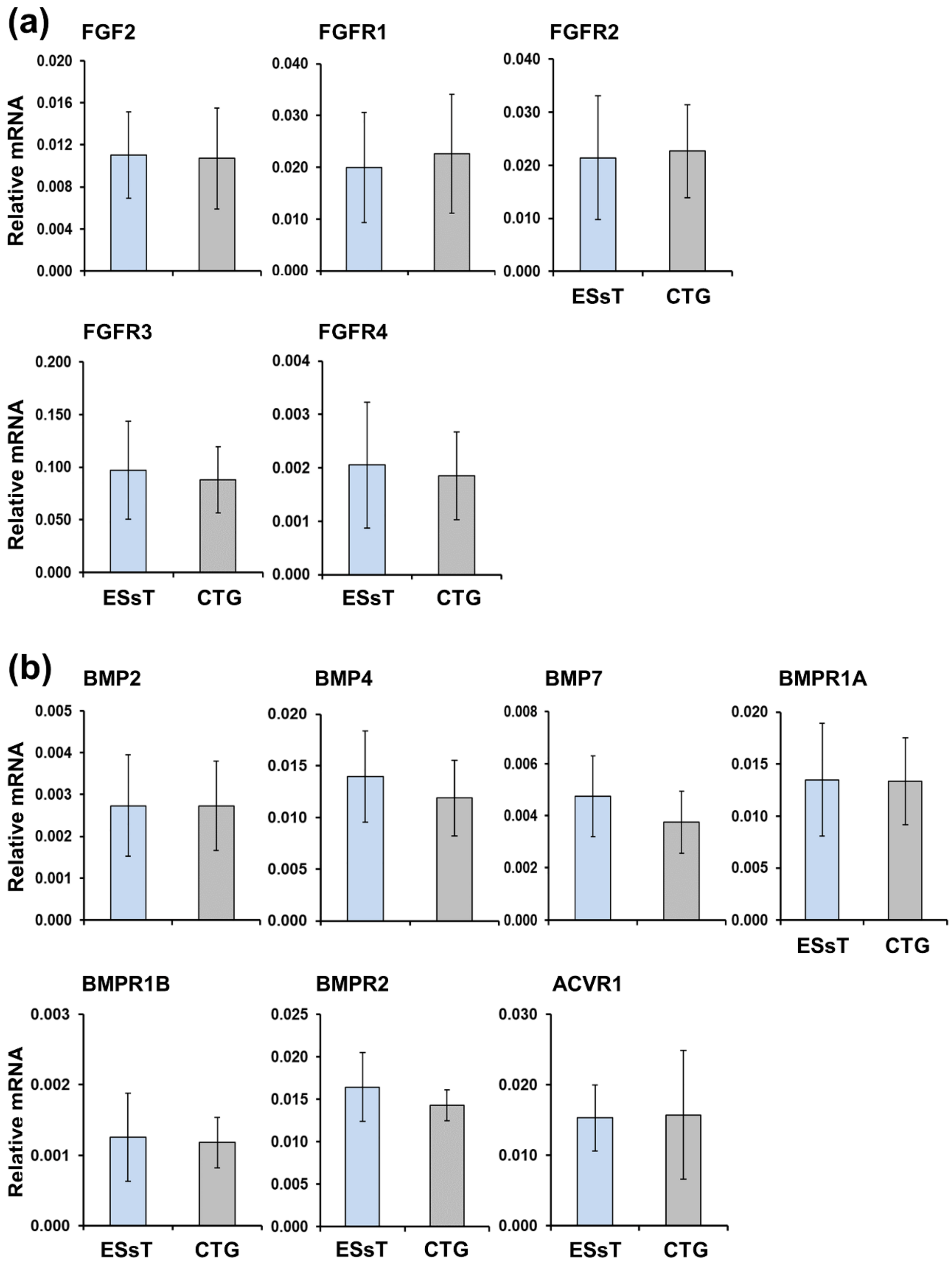

Figure S1. No significant changes in the expression of genes encoding (**a**) FGF-2 and its receptors, and (**b**) BMP-2, -4, -7 and their receptors in ESsT and CTG samples. qRT-PCR analyses of (**a**) FGF2, FGFR1, FGFR2, FGFR3, FGFR4, and (**b**) BMP2, BMP4, BMP7, BMPR1A, BMPR1B, BMPR2, ACVR1 (encoding activin A receptor, type I) transcripts normalized to GAPDH in the ESsT and CTG samples. Means ± SD for 6 patients are shown.

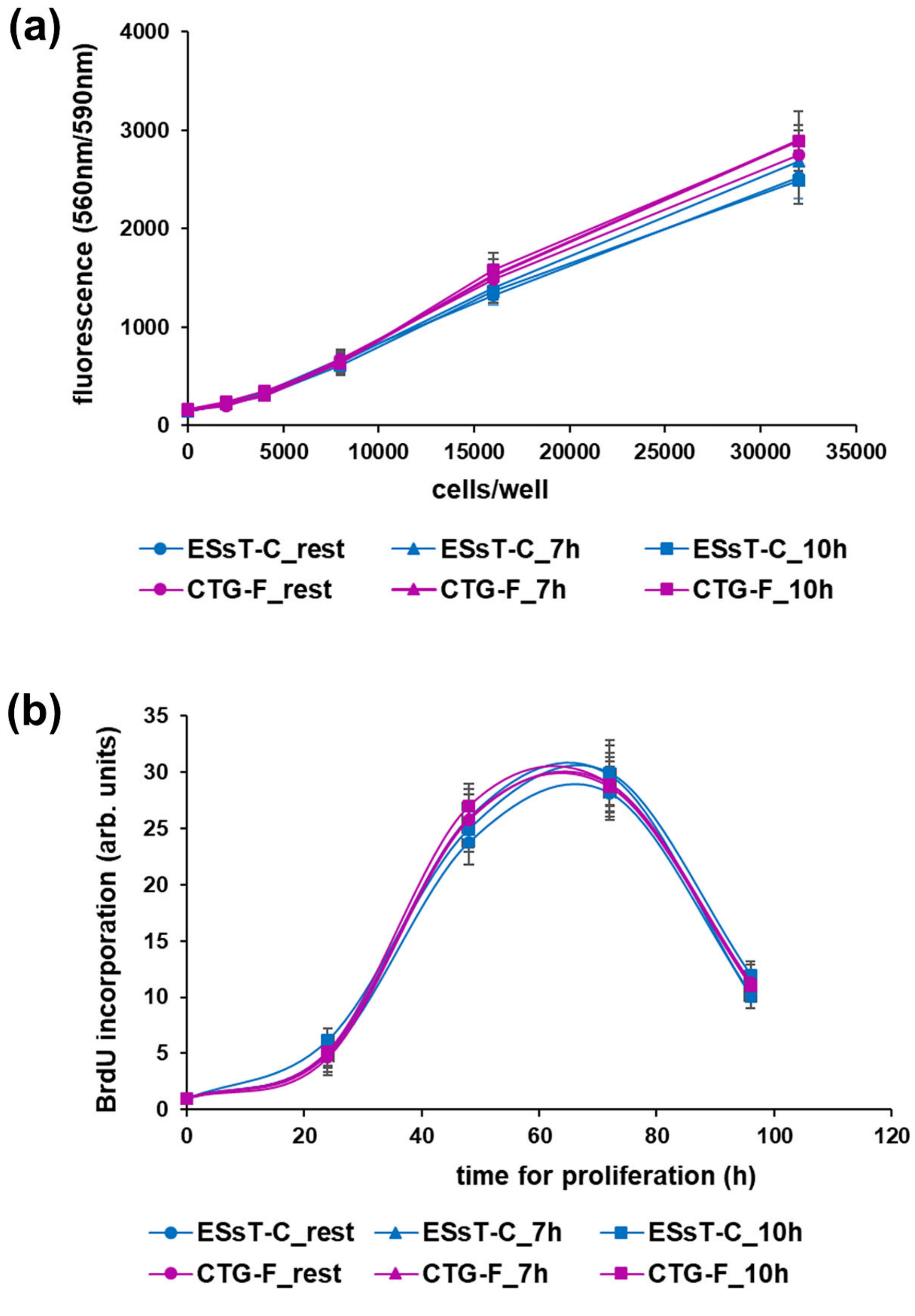

Figure S2. No significant changes in cell viability and proliferation of primary ESsT-Cs and CTG-Fs subjected to intermittent equibiaxial cyclic strain. ESsT-Cs and CTG-Fs cultured on fibronectin-coated silicone membranes were left at rest (no mechanical stimulation) or cyclically strained as described in Figure 3a in the main text, and then subjected to the CellTiter-Blue cell viability assay (**a**) and the BrdU Cell Proliferation ELISA (**b**) according to the manufacturers' protocols.

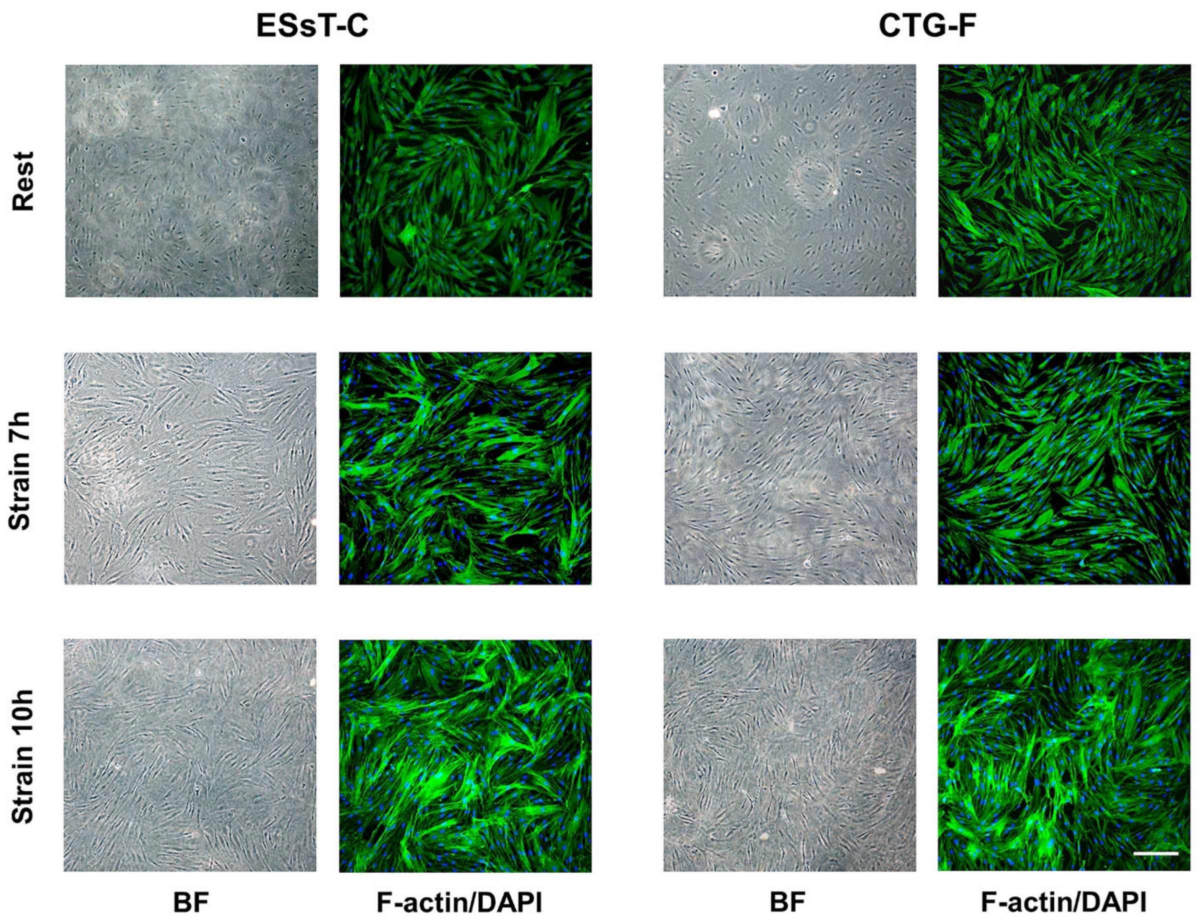

Figure S3. Morphological appearance and actin stress fiber formation in primary ESsT-Cs and CTG-Fs subjected to intermittent equibiaxial cyclic strain. Primary ESsT-Cs and CTG-Fs cultured on fibronectin-coated silicone membranes were left at rest (no mechanical stimulation) or cyclically strained, fixed, and stained with Alexa Fluor 488-labeled phalloidin (green) for actin stress fibers. The cell nuclei were localized via DAPI co-stain (blue); a bright field (BF) image is also shown. Scale bar, 500 µm.

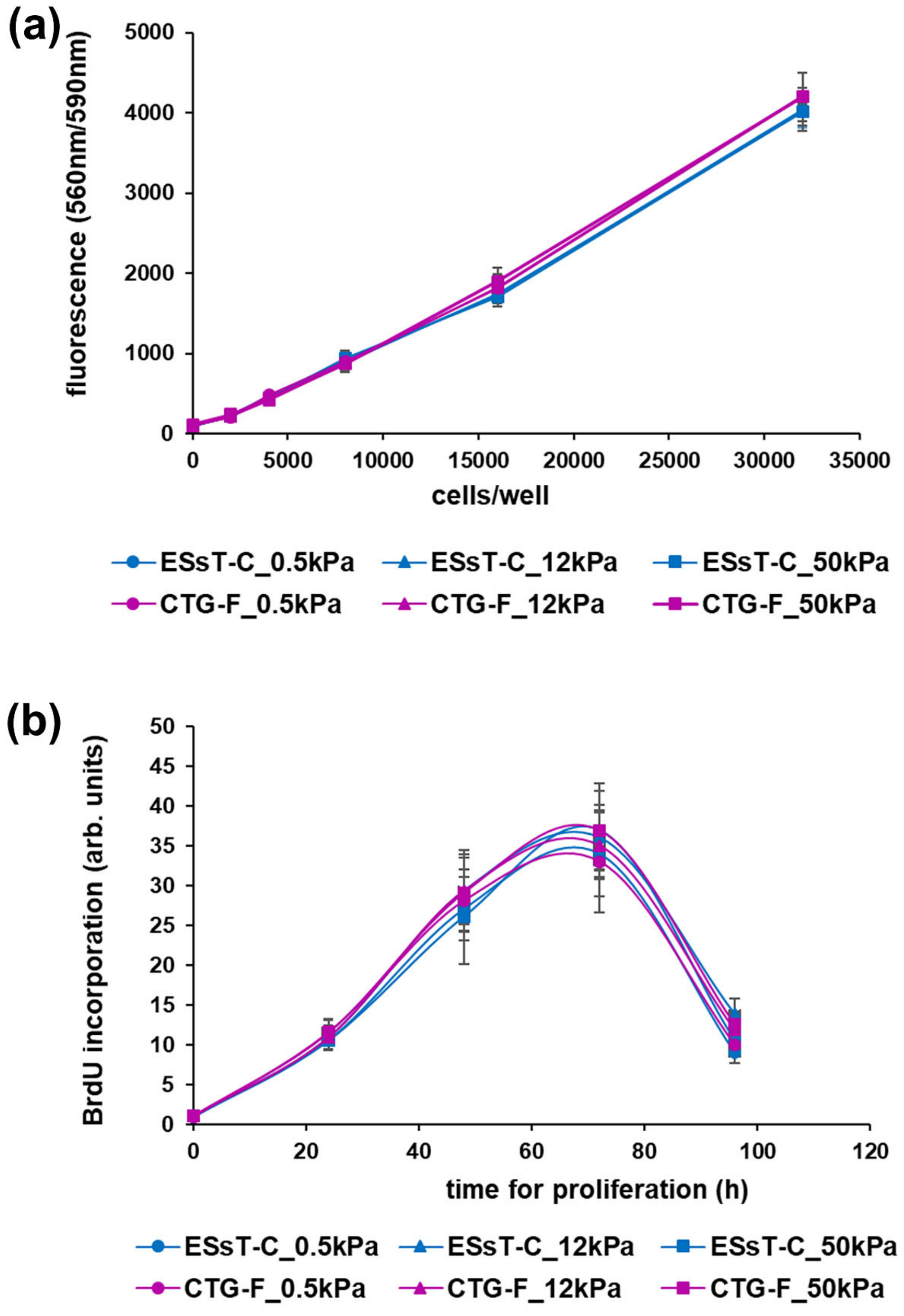

Figure S4. No significant changes in cell viability and proliferation of primary ESsT-Cs and CTG-Fs grown on soft (0.5 kPa) or stiff (12 or 50 kPa) matrices. ESsT-Cs and CTG-Fs were cultured on fibronectin-coated polyacrylamide hydrogels of defined stiffness as described in Figure 5a in the main text, and then subjected to the CellTiter-Blue Cell Viability assay (**a**) and the BrdU Cell Proliferation ELISA (**b**) according to the manufacturers' protocols.

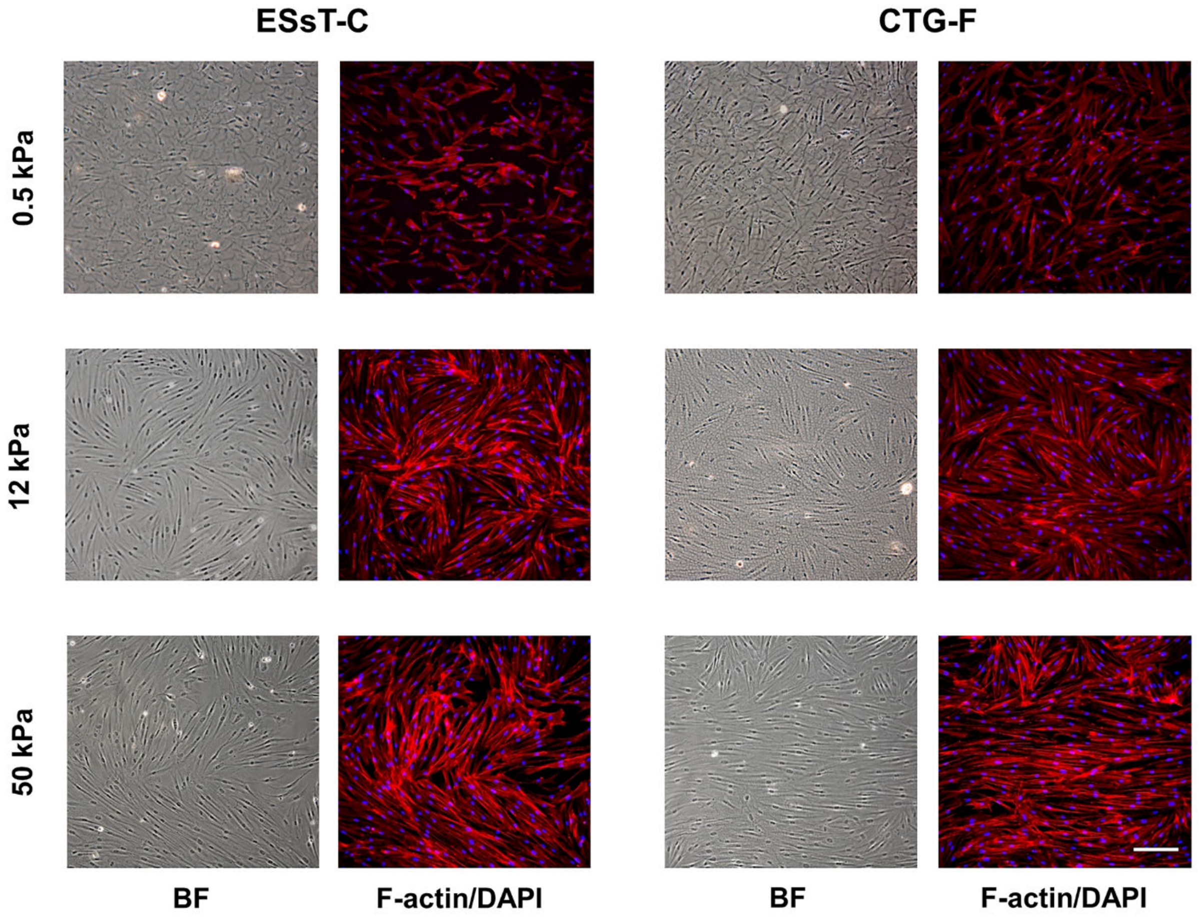

Figure S5. Morphological appearance and actin stress fiber formation in primary ESsT-Cs and CTG-Fs grown on soft (0.5 kPa) or stiff (12 or 50 kPa) matrices. Primary ESsT-Cs and CTG-Fs were cultured on fibronectin-coated polyacrylamide hydrogels of defined stiffness as described in Figure 5a, fixed, and stained with tetramethylrhodamine (TRITC)-labeled phalloidin (red) for actin stress fibers. The cell nuclei were localized via DAPI co-stain (blue); a bright field (BF) image is also shown. Scale bar, 500 μm.

Table S1: Primer sequences for osteogenesis marker genes.

Gene symbol	Primer pair (fwd/rev)
COL1A1	5′-GAAGGGACACAGAGGTTTCAG-3'
	5′-TAGCACCATCATTTCCACGA-3'
SPP1	5′-ATGACACTGATGATTCTCACCA-3'
	5′-GCATCAGGGTACTGGATGTC-3'
RUNX2	5′-AGACCAACAGAGTCATTTAAGGC-3'
	5′-GGTGTCACTGTGCTGAAGAG-3'
ALPL	5′-TGGCAACTCTATCTTTGGTCTG-3'
	5′-TTGTTGTGAGCATAGTCCACC-3'
DLX5	5′-TTCCAAGCTCCGTTCCAGAC-3'
	5′-GAATCGGTAGCTGAAGACTCG-3'
IBSP	5′-GGAATGGCCTGTGCTTTCTC-3'
	5′-AGTCACTACTGCCCTGAACTG-3'
BGLAP2	5′-GTGCAGAGTCCAGCAAAGGT-3'
	5′-TCAGCCAACTCGTCACAGTC-3'
PHEX	5′-TTTCTTCCGGTTCGCTTGTGA-3'
	5′-AGTTCCTTCAACTTGAGGTCAAC-3'
GAPDH*	5′-ATCAAGAAGGTGGTGAAGCAG-3'
	5′-TCGTTGTCATACCAGGAAATGAG-3'

*reference gene used for normalization in all qPCR analyses

Table S2: Primer sequences for genes encoding extracellular matrix proteins.

Gene symbol	Primer pair (fwd/rev)
COL1A2	5′-TGGACCTCCTGGTAATCCTG-3'
	5′-GCTCACCAACAAGTCCTCTG-3'
COL3A1	5′-AAGGAAATGATGGTGCTCCTG-3'
	5′-AGCCTTGTAATCCTTGTGGAC-3'
POSTN	5′-ACTCCTCTATCCAGCAGACAC-3'
	5′-TAATTGGCTTATAGACAGTCACGG-3'
FN1	5′-TGCAGGTCCAGATCAAACAG-3'
	5′-TCCACATCAGTGAATGCCAG-3'

VIM	5′-TGAACCTGAGGGAAACTAATCTG-3'
	5′-TCGTTGATAACCTGTCCATCTC-3'
TNC	5′-GAGGGTGACCACCACACGCTT-3'
	5′-CAAGGCAGTGGTGTCTGGACATC-3'

Table S3: Primer sequences encoding isoforms of FGF and BMP and their receptors.

Gene symbol	Primer pair (fwd/rev)
FGF2	5′-ACATCAAGCTACAACTTCAAGC-3'
	5′-CCGTAACACATTTAGAAGCCAG-3'
FGFR1	5′-CTGTGAAGATGTTGAAGTCGG-3'
	5′-ACATACAAGGGACCATCCTG-3'
FGFR2	5′-CAAGAGATAAGCTGACACTGGG-3'
	5′-CATCATCTTTCAACATCTTCACGG-3'
FGFR3	5′-AAGATGCTGAAAGACGATGCC-3'
	5′-TACTCCACCAGCACGTACAG-3'
FGFR4	5′-TGAGTCTAGATCTACCTCTCGAC-3'
	5′-TCAGAGGCGTTGTCTTTGAG-3'
BMP2	5′-CCACCATGAAGAATCTTTGGA-3'
	5′-AGCATCTTGCATCTGTTCTC-3'
BMP4	5′-CACCACGAAGAACATCTGGAG-3'
	5′-AATGTTTATACGGTGGAAGCCC-3'
BMP7	5′-GTCAACCTCGTGGAACATGAC-3'
	5′-AAGAGATCCGATTCCCTGCC-3'
BMPR1A	5′-TCGTTGTATCACAGGAGGGA-3'
	5′-ACTGCTCGTAGACATTCATCAC-3'
BMPR1B	5′-TGTGTATCAGGAGGTATAGTGG-3'
	5′-CTTAGACACTCATCACTGCTC-3'
BMPR2	5′-AACACCACTCAGTCCACCTC-3'
	5′-CGGTCTCCTGTCAACATTCTG-3'
ACVR1	5′-TGAGCAATGGTATAGTGGAGG-3'
	5′-AGGTTAATGTCGGGTCTGAG-3'

Table S4: Primer sequences encoding isoforms of TGF-β and IGF and their receptors.

Gene symbol	Primer pair (fwd/rev)
TGFB1	5′-AACCCACAACGAAATCTATGAC-3'
	5′-GGAATTGTTGCTGTATTTCTGG-3'
TGFB2	5′-GATTTGCAGGTATTGATGGCAC-3'
	5′-TTTCTAAAGCAATAGGCCGCA-3'
TGFB3	5′-GAGCTCTTCCAGATCCTTCG-3'
	5′-TTTCTAGACCTAAGTTGGACTCTC-3'
TGFBR1	5′-TAGTATTCTGGGAAATTGCTCGAC-3'
	5′-CTCTCAAGGCTTCACAGCTC-3'
TGFBR2	5′-GTGGCTGTATGGAGAAAGAATGAC-3'
	5′-AACACATGAAGAAAGTCTCACCAG-3'
IGF1	5′-TTTATTTCAACAAGCCCACAGG-3'
	5′-GCTGATACTTCTGGGTCTTGG-3'
IGF2	5′-CCTGGACAATCAGACGAATTCTC-3'
	5′-CATTGGTGTCTGGAAGCCG-3'
IGFR1	5′-GAGCCTCCTGTGAAAGTGAC-3'
	5′-CATCCTGCCCATCATACTCTG-3'
IGF2R	5′-CACCATTCCCAAACTCACAG-3'
	5′-AATATAGGATGAACCTCCGCTC-3'
